# 1-(2-Hy­droxy-4-meth­oxy­phen­yl)-3-(4-methyl­phen­yl)prop-2-en-1-one

**DOI:** 10.1107/S1600536811007586

**Published:** 2011-03-09

**Authors:** G. B. Thippeswamy, D. Vijay Kumar, B. S. Jayashree, M. A. Sridhar, J. Shashidhara Prasad

**Affiliations:** a Department of Studies in Physics, Manasagangotri, University of Mysore, Mysore 570 006, India; b Department of Pharmaceutical Chemistry, Manipal college of Pharmaceutical Sciences, Manipal 576 104, India

## Abstract

The mol­ecule of the title compound, C_17_H_16_O_3_, exists in the *E* conformation with respect to the central C=C bond, is almost planar(r.m.s. deviation = 0.003 Å) and has an intra­molecular O—H⋯O hydrogen bond, which generates an *S*(6) ring. In the crystal, mol­ecules are linked by C—H⋯O inter­actions.

## Related literature

For the biological activity of compounds with a chalcone backbone, see: Jayashree *et al.* (2009 ▶); Epifano *et al.* (2007 ▶); Onyilagna *et al.* (1997 ▶); Satyanarayana *et al.* (2004 ▶); Deshpande *et al.* (1999 ▶); Hsieh *et al.* (2000 ▶); Khatib *et al.* (2005 ▶); Barford *et al.* (2002 ▶); Nielsen *et al.* (1995 ▶); Severi *et al.* (1998 ▶); Siva Kumar *et al.* (2007 ▶). For a related structure, see: Thippeswamy *et al.* (2010 ▶). For puckering parameters, see: Cremer & Pople (1975 ▶). 
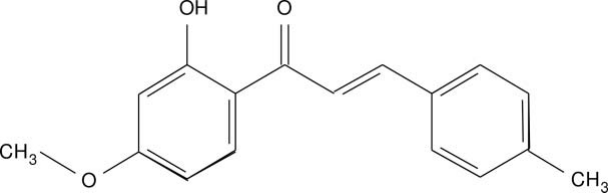


## Experimental

### 

#### Crystal data

C_17_H_16_O_3_
*M* *_r_* = 268.30
Monoclinic, 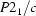

*a* = 11.340 (2) Å
*b* = 6.8350 (7) Å
*c* = 20.449 (4) Å
β = 117.710 (4)°
*V* = 1403.2 (4) Å^3^
*Z* = 4
Mo *K*α radiation
μ = 0.09 mm^−1^
*T* = 293 K
0.26 × 0.24 × 0.22 mm

#### Data collection

MacScience DIPLabo 32001 diffractometer
4137 measured reflections
2346 independent reflections
1502 reflections with *I* > 2σ(*I*)
*R*_int_ = 0.029

#### Refinement

*R*[*F* ^2^ > 2σ(*F* ^2^)] = 0.057
*wR*(*F* ^2^) = 0.184
*S* = 1.05
2346 reflections
184 parameters
H-atom parameters constrained
Δρ_max_ = 0.15 e Å^−3^
Δρ_min_ = −0.15 e Å^−3^

### 

Data collection: *XPRESS* (MacScience, 2002 ▶); cell refinement: *SCALEPACK* (Otwinowski & Minor, 1997 ▶); data reduction: *DENZO* (Otwinowski & Minor, 1997 ▶) and *SCALEPACK*; program(s) used to solve structure: *SHELXS97* (Sheldrick, 2008 ▶); program(s) used to refine structure: *SHELXL97* (Sheldrick, 2008 ▶); molecular graphics: *PLATON* (Spek, 2009 ▶) and *ORTEPII* (Johnson, 1976 ▶); software used to prepare material for publication: *PLATON*.

## Supplementary Material

Crystal structure: contains datablocks global, I. DOI: 10.1107/S1600536811007586/jh2240sup1.cif

Structure factors: contains datablocks I. DOI: 10.1107/S1600536811007586/jh2240Isup2.hkl

Additional supplementary materials:  crystallographic information; 3D view; checkCIF report

## Figures and Tables

**Table 1:** Hydrogen-bond geometry (Å, °)

*D*—H⋯*A*	*D*—H	H⋯*A*	*D*⋯*A*	*D*—H⋯*A*
O18—H18⋯O11	0.82	1.77	2.502 (3)	148
C13—H13⋯O18^i^	0.93	2.56	3.282 (3)	135

Symmetry code: (i) 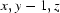.

**Fig. 1.**
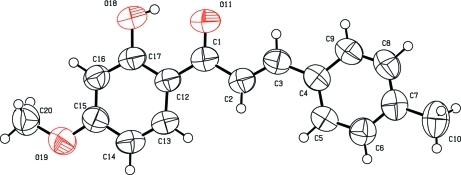
Crystal structure of the title compound with 50% probability displacement ellipsoids.

**Fig. 2.**
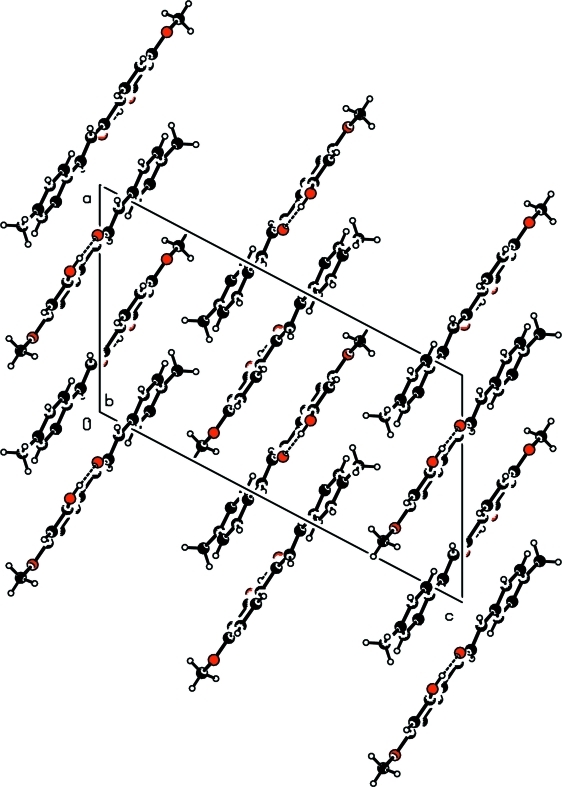
The packing of the title compound, viewed down the b axis.

## supplementary crystallographic information

### Comment

Chalcones (1,3-diarylpropenones) are well known intermediates for the synthesis of various heterocyclic compounds. The compounds with chalcone backbone have been reported to possess various biological activities such as anti-oxidant (Jayashree *et al.*, 2009), anti-inflammatory (Hsieh *et al.*, 2000), anti-cancer (Epifano *et al.*, 2007), anti-hyperglycemic (Satyanarayana *et al.*, 2004), anti-viral (Onyilagna *et al.*, 1997), anti-leishmanial (Nielsen *et al.*,1995), anti-tubercular (Siva Kumar *et al.*, 2007), immunomodulatory (Barford *et al.*, 2002), inhibition of various enzymes like leukotriene B (Deshpande *et al.*, 1999), tyrosinase kinase (Khatib *et al.*, 2005) and aldose reductase (Severi *et al.*, 1998) *etc*. The presence of a reactive alph, beta-unsaturated ketone function in chalcones is found to be responsible for their activity. In the present communication, we report the synthesis and crystal structure of substituted 2-hydroxy-chalcone. In the title compound, C17H16O3, the dihedral angle between the ring systems is 9.57 (13)°. The central prop-2-en-1-oneunit is planar (r.m.s. deviation = 0.003 Å) and is oriented at a dihedral angle of 2.46 (10)° with respect to the methoxyphenyl ring and at 7.46 (10)° with respect to the methylphenyl ring. The angles C2—C1—O11, C12—C1—O11 and C2—C1—C12 are 118.5 (3)°, 119.9 (2)° and 121.5 (2)° respectively which indicate that the position of C1 atom is nearly in trigonal geometry. The bond lengths and bond angles of the molecule are comparable with the values reported for 1-(2-hydroxy-5-methylphenyl)-3-(3-methylthiophen-2-yl) prop-2-en-1-one (Thippeswamy *et al.*,2010). The atoms C4, C7 in methylphenyl ring deviate by -0.018 (2) Å, -0.014 (3) Å, and the atoms C12, C15 in methoxyphenyl ring deviate by -0.002 (2) Å, -0.003 (3)Å respectively from Cremer and Pople plane (Cremer *et al.*,1975) which show that the two six-membered rings are in planar conformation. The packing of the molecules is characterized by intramolecular hydrogen bond of type O–H–O.

### Experimental

The title compound was prepared by dissolving 2-hydroxy-4- methoxyacetophenone 0.05 m mol in 15 ml of ethanol taken in a conical flask. To this 5 ml of 20° aqueous sodium hydroxide was added and kept for stirring at room temperature. To this mixture, 4-methylbenzaldehyde 0.05 m mol was added and continued stirring till the completion of reaction. The progress of the reaction was monitored by TLC using n-hexane and ethyl acetate as solvent system. After completion of the reaction, the mixture was poured into ice cold water, mixed properly and acidified with dilute hydrochloric acid. The title compound separates as precipitate which was collected by filtration and crystallized from methanol. The compound was chafacterized by spectroscope technique. The IR spectrum was recorded in KBr on FTIR-8400 (Shimadzu). The 1H NMR spectrum was recorded in CdCl3 solution at 400 MHz on AMX 400 MHz High Resolution Multinuclear FT-NMR Spectrometer (Bruker) with tetramethylsilane (TMS) as internal standard.

### Crystal data

**Table:**

C_17_H_16_O_3_	*F*(000) = 568
*M**_r_* = 268.30	*D*_x_ = 1.270 Mg m^−^^3^
Monoclinic, *P*2_1_/*c*	Mo *K*α radiation, λ = 0.71073 Å
Hall symbol: -P 2ybc	Cell parameters from 4137 reflections
*a* = 11.340 (2) Å	θ = 2.2–25.0°
*b* = 6.8350 (7) Å	µ = 0.09 mm^−^^1^
*c* = 20.449 (4) Å	*T* = 293 K
β = 117.710 (4)°	Block, yellow
*V* = 1403.2 (4) Å^3^	0.26 × 0.24 × 0.22 mm
*Z* = 4	

### Data collection

**Table:**

MacScience DIPLabo 32001 diffractometer	1502 reflections with *I* > 2σ(*I*)
Radiation source: fine-focus sealed tube	*R*_int_ = 0.029
graphite	θ_max_ = 25.0°, θ_min_ = 2.2°
Detector resolution: 10.0 pixels mm^-1^	*h* = −13→13
ω scans	*k* = −7→7
4137 measured reflections	*l* = −24→24
2346 independent reflections	

### Refinement

**Table:**

Refinement on *F*^2^	Secondary atom site location: difference Fourier map
Least-squares matrix: full	Hydrogen site location: inferred from neighbouring sites
*R*[*F*^2^ > 2σ(*F*^2^)] = 0.057	H-atom parameters constrained
*wR*(*F*^2^) = 0.184	*w* = 1/[σ^2^(*F*_o_^2^) + (0.1052*P*)^2^ + 0.0529*P*]
*S* = 1.05	(Δ/σ)_max_ = 0.010
2346 reflections	Δρ_max_ = 0.15 e Å^−^^3^
184 parameters	Δρ_min_ = −0.15 e Å^−^^3^
0 restraints	Extinction correction: *SHELXL97* (Sheldrick, 2008), Fc^*^=kFc[1+0.001xFc^2^λ^3^/sin(2θ)]^-1/4^
Primary atom site location: structure-invariant direct methods	Extinction coefficient: 0.032 (8)

### Special details

**Table:**

Geometry. Bond distances, angles *etc*. have been calculated using the rounded fractional coordinates. All su's are estimated from the variances of the (full) variance-covariance matrix. The cell e.s.d.'s are taken into account in the estimation of distances, angles and torsion angles
Refinement. Refinement on *F*^2^ for ALL reflections except those flagged by the user for potential systematic errors. Weighted *R*-factors *wR* and all goodnesses of fit *S* are based on *F*^2^, conventional *R*-factors *R* are based on *F*, with *F* set to zero for negative *F*^2^. The observed criterion of *F*^2^ > σ(*F*^2^) is used only for calculating -*R*-factor-obs *etc*. and is not relevant to the choice of reflections for refinement. *R*-factors based on *F*^2^ are statistically about twice as large as those based on *F*, and *R*-factors based on ALL data will be even larger.

### Fractional atomic coordinates and isotropic or equivalent isotropic displacement parameters (Å^2^)

**Table:**

	*x*	*y*	*z*	*U*_iso_*/*U*_eq_	
O11	0.22698 (19)	0.0248 (2)	0.50576 (11)	0.1003 (8)	
O18	0.4503 (2)	0.1776 (2)	0.58182 (12)	0.1010 (8)	
O19	0.82822 (19)	−0.2381 (3)	0.68536 (10)	0.0952 (8)	
C1	0.2778 (3)	−0.1418 (3)	0.51232 (13)	0.0769 (9)	
C2	0.1894 (3)	−0.3084 (4)	0.47598 (14)	0.0781 (9)	
C3	0.0588 (3)	−0.2910 (4)	0.44711 (13)	0.0788 (9)	
C4	−0.0439 (2)	−0.4376 (4)	0.40885 (12)	0.0745 (9)	
C5	−0.0165 (3)	−0.6329 (4)	0.40093 (13)	0.0771 (9)	
C6	−0.1187 (3)	−0.7615 (4)	0.36199 (14)	0.0829 (10)	
C7	−0.2504 (3)	−0.7042 (4)	0.33000 (13)	0.0853 (10)	
C8	−0.2767 (3)	−0.5123 (5)	0.34017 (15)	0.0941 (11)	
C9	−0.1758 (3)	−0.3830 (4)	0.37904 (14)	0.0876 (10)	
C10	−0.3608 (3)	−0.8452 (5)	0.28614 (16)	0.1111 (14)	
C12	0.4201 (2)	−0.1668 (3)	0.55578 (12)	0.0695 (8)	
C13	0.4837 (3)	−0.3491 (3)	0.56897 (14)	0.0777 (9)	
C14	0.6169 (3)	−0.3701 (3)	0.61167 (14)	0.0833 (10)	
C15	0.6964 (3)	−0.2045 (4)	0.64370 (14)	0.0771 (9)	
C16	0.6383 (3)	−0.0211 (3)	0.63231 (14)	0.0775 (10)	
C17	0.5025 (3)	−0.0035 (3)	0.58930 (13)	0.0735 (9)	
C20	0.9130 (3)	−0.0751 (4)	0.71775 (17)	0.1034 (12)	
H2	0.22610	−0.42800	0.47330	0.0940*	
H3	0.02810	−0.16830	0.45180	0.0950*	
H5	0.07130	−0.67620	0.42210	0.0920*	
H6	−0.09840	−0.89080	0.35710	0.0990*	
H8	−0.36460	−0.47010	0.32020	0.1130*	
H9	−0.19680	−0.25530	0.38550	0.1050*	
H10A	−0.42770	−0.83800	0.30230	0.1670*	
H10B	−0.32560	−0.97570	0.29340	0.1670*	
H10C	−0.39960	−0.81220	0.23470	0.1670*	
H13	0.43250	−0.45980	0.54770	0.0930*	
H14	0.65550	−0.49360	0.61970	0.1000*	
H16	0.69040	0.08900	0.65340	0.0930*	
H18	0.36950	0.17260	0.55550	0.1510*	
H20A	0.90600	0.01340	0.67960	0.1550*	
H20B	1.00340	−0.11930	0.74500	0.1550*	
H20C	0.88710	−0.00910	0.75050	0.1550*	

### Atomic displacement parameters (Å^2^)

**Table:**

	*U*^11^	*U*^22^	*U*^33^	*U*^12^	*U*^13^	*U*^23^
O11	0.0982 (14)	0.0672 (11)	0.1254 (16)	0.0151 (9)	0.0435 (12)	0.0038 (10)
O18	0.1048 (15)	0.0578 (10)	0.1353 (16)	0.0064 (9)	0.0516 (12)	0.0001 (9)
O19	0.0834 (14)	0.0929 (13)	0.1058 (14)	0.0051 (10)	0.0411 (11)	−0.0085 (10)
C1	0.0879 (18)	0.0692 (14)	0.0771 (15)	0.0051 (12)	0.0414 (14)	0.0029 (11)
C2	0.0831 (18)	0.0705 (14)	0.0819 (16)	0.0012 (12)	0.0393 (13)	−0.0014 (11)
C3	0.0831 (19)	0.0788 (15)	0.0731 (15)	0.0096 (13)	0.0352 (14)	0.0036 (12)
C4	0.0741 (17)	0.0851 (16)	0.0634 (14)	0.0081 (12)	0.0313 (12)	0.0052 (11)
C5	0.0742 (16)	0.0832 (16)	0.0737 (15)	0.0129 (12)	0.0343 (13)	0.0039 (12)
C6	0.0848 (19)	0.0857 (16)	0.0788 (16)	−0.0035 (14)	0.0385 (14)	−0.0054 (13)
C7	0.0818 (19)	0.1053 (19)	0.0654 (15)	−0.0058 (15)	0.0314 (13)	0.0042 (13)
C8	0.0705 (17)	0.114 (2)	0.0881 (18)	0.0105 (15)	0.0288 (14)	0.0126 (16)
C9	0.0760 (18)	0.0920 (17)	0.0909 (18)	0.0163 (14)	0.0355 (15)	0.0095 (14)
C10	0.096 (2)	0.141 (3)	0.0857 (19)	−0.0278 (19)	0.0334 (16)	−0.0083 (18)
C12	0.0817 (17)	0.0599 (12)	0.0743 (14)	0.0020 (11)	0.0426 (13)	0.0004 (10)
C13	0.0884 (18)	0.0621 (13)	0.0843 (16)	0.0032 (11)	0.0415 (14)	−0.0045 (11)
C14	0.096 (2)	0.0649 (14)	0.0892 (17)	0.0089 (12)	0.0433 (15)	−0.0031 (12)
C15	0.0809 (18)	0.0813 (15)	0.0758 (15)	0.0040 (13)	0.0420 (14)	−0.0022 (12)
C16	0.0836 (19)	0.0677 (14)	0.0885 (17)	−0.0030 (12)	0.0461 (15)	−0.0061 (12)
C17	0.0923 (19)	0.0577 (13)	0.0832 (15)	0.0049 (11)	0.0514 (14)	0.0018 (11)
C20	0.087 (2)	0.112 (2)	0.107 (2)	−0.0097 (17)	0.0417 (17)	−0.0209 (17)

### Geometric parameters (Å, °)

**Table:**

O11—C1	1.255 (3)	C14—C15	1.405 (4)
O18—C17	1.350 (3)	C15—C16	1.385 (4)
O19—C15	1.352 (4)	C16—C17	1.378 (5)
O19—C20	1.419 (4)	C2—H2	0.9300
O18—H18	0.8200	C3—H3	0.9300
C1—C2	1.470 (4)	C5—H5	0.9300
C1—C12	1.445 (4)	C6—H6	0.9300
C2—C3	1.320 (5)	C8—H8	0.9300
C3—C4	1.458 (4)	C9—H9	0.9300
C4—C9	1.379 (4)	C10—H10A	0.9600
C4—C5	1.397 (4)	C10—H10B	0.9600
C5—C6	1.375 (4)	C10—H10C	0.9600
C6—C7	1.380 (5)	C13—H13	0.9300
C7—C8	1.382 (4)	C14—H14	0.9300
C7—C10	1.502 (4)	C16—H16	0.9300
C8—C9	1.370 (5)	C20—H20A	0.9600
C12—C17	1.412 (3)	C20—H20B	0.9600
C12—C13	1.402 (3)	C20—H20C	0.9600
C13—C14	1.356 (5)		
			
O11···O18	2.502 (3)	H2···C5	2.8200
O18···O11	2.502 (3)	H2···C13	2.7100
O18···C13^i^	3.282 (3)	H2···H5	2.3100
O11···H3	2.3900	H2···H13	2.1300
O11···H9^ii^	2.8700	H3···O11	2.3900
O11···H5^i^	2.7200	H3···H9	2.3400
O11···H18	1.7700	H5···O11^v^	2.7200
O18···H13^i^	2.5600	H5···C2	2.8200
C1···C7^iii^	3.540 (4)	H5···H2	2.3100
C3···C5^iii^	3.398 (4)	H6···H10B	2.3600
C3···C4^iii^	3.551 (4)	H8···H10A	2.5900
C4···C4^iii^	3.488 (3)	H8···C10^viii^	2.9800
C4···C3^iii^	3.551 (4)	H9···H3	2.3400
C5···C3^iii^	3.398 (4)	H9···O11^ii^	2.8700
C6···C20^iv^	3.590 (5)	H10A···H8	2.5900
C7···C1^iii^	3.540 (4)	H10A···C12^iii^	2.8600
C10···C12^iii^	3.596 (4)	H10A···C17^iii^	2.9200
C12···C10^iii^	3.596 (4)	H10B···H6	2.3600
C13···O18^v^	3.282 (3)	H13···O18^v^	2.5600
C20···C6^iv^	3.590 (5)	H13···C2	2.6600
C1···H18	2.3700	H13···H2	2.1300
C2···H13	2.6600	H16···C20	2.5000
C2···H5	2.8200	H16···H20A	2.3100
C4···H20C^vi^	2.9800	H16···H20C	2.2900
C5···H2	2.8200	H18···O11	1.7700
C5···H20C^vi^	2.9100	H18···C1	2.3700
C6···H20C^vi^	2.9600	H20A···C16	2.7300
C7···H20C^vi^	3.0900	H20A···H16	2.3100
C10···H8^vii^	2.9800	H20C···C16	2.7300
C12···H10A^iii^	2.8600	H20C···H16	2.2900
C13···H2	2.7100	H20C···C4^ix^	2.9800
C16···H20A	2.7300	H20C···C5^ix^	2.9100
C16···H20C	2.7300	H20C···C6^ix^	2.9600
C17···H10A^iii^	2.9200	H20C···C7^ix^	3.0900
C20···H16	2.5000		
			
C15—O19—C20	118.1 (2)	C3—C2—H2	119.00
C17—O18—H18	109.00	C2—C3—H3	116.00
O11—C1—C2	118.5 (3)	C4—C3—H3	116.00
O11—C1—C12	119.9 (2)	C4—C5—H5	120.00
C2—C1—C12	121.5 (2)	C6—C5—H5	120.00
C1—C2—C3	121.1 (3)	C5—C6—H6	119.00
C2—C3—C4	128.9 (3)	C7—C6—H6	119.00
C3—C4—C9	118.9 (3)	C7—C8—H8	119.00
C5—C4—C9	117.5 (3)	C9—C8—H8	119.00
C3—C4—C5	123.6 (3)	C4—C9—H9	119.00
C4—C5—C6	120.3 (3)	C8—C9—H9	119.00
C5—C6—C7	121.9 (3)	C7—C10—H10A	110.00
C6—C7—C8	117.4 (3)	C7—C10—H10B	109.00
C6—C7—C10	121.3 (3)	C7—C10—H10C	109.00
C8—C7—C10	121.4 (3)	H10A—C10—H10B	109.00
C7—C8—C9	121.3 (3)	H10A—C10—H10C	109.00
C4—C9—C8	121.6 (3)	H10B—C10—H10C	109.00
C1—C12—C13	123.4 (2)	C12—C13—H13	119.00
C1—C12—C17	120.2 (2)	C14—C13—H13	119.00
C13—C12—C17	116.4 (2)	C13—C14—H14	120.00
C12—C13—C14	122.5 (2)	C15—C14—H14	120.00
C13—C14—C15	119.8 (2)	C15—C16—H16	120.00
O19—C15—C16	124.1 (3)	C17—C16—H16	120.00
C14—C15—C16	119.9 (3)	O19—C20—H20A	109.00
O19—C15—C14	116.0 (3)	O19—C20—H20B	109.00
C15—C16—C17	119.3 (2)	O19—C20—H20C	110.00
O18—C17—C16	117.0 (2)	H20A—C20—H20B	109.00
C12—C17—C16	122.1 (2)	H20A—C20—H20C	110.00
O18—C17—C12	120.9 (3)	H20B—C20—H20C	109.00
C1—C2—H2	119.00		
			
C20—O19—C15—C16	−1.9 (4)	C5—C6—C7—C10	178.6 (3)
C20—O19—C15—C14	178.8 (3)	C10—C7—C8—C9	−178.8 (3)
C12—C1—C2—C3	168.4 (3)	C6—C7—C8—C9	1.5 (4)
O11—C1—C12—C13	176.6 (3)	C7—C8—C9—C4	0.9 (4)
C2—C1—C12—C13	−2.5 (4)	C17—C12—C13—C14	0.2 (4)
C2—C1—C12—C17	179.3 (3)	C1—C12—C13—C14	−178.0 (3)
O11—C1—C12—C17	−1.7 (4)	C13—C12—C17—O18	−178.0 (2)
O11—C1—C2—C3	−10.7 (4)	C13—C12—C17—C16	0.4 (4)
C1—C2—C3—C4	179.5 (2)	C1—C12—C17—O18	0.4 (4)
C2—C3—C4—C9	−176.0 (3)	C1—C12—C17—C16	178.8 (3)
C2—C3—C4—C5	4.5 (4)	C12—C13—C14—C15	−0.9 (4)
C3—C4—C5—C6	−177.9 (3)	C13—C14—C15—C16	0.8 (4)
C5—C4—C9—C8	−2.9 (4)	C13—C14—C15—O19	−179.9 (3)
C9—C4—C5—C6	2.6 (4)	O19—C15—C16—C17	−179.4 (3)
C3—C4—C9—C8	177.6 (3)	C14—C15—C16—C17	−0.2 (4)
C4—C5—C6—C7	−0.4 (4)	C15—C16—C17—O18	178.0 (3)
C5—C6—C7—C8	−1.7 (4)	C15—C16—C17—C12	−0.5 (4)

Symmetry codes: (i) *x*, *y*+1, *z*; (ii) −*x*, −*y*, −*z*+1; (iii) −*x*, −*y*−1, −*z*+1; (iv) −*x*+1, −*y*−1, −*z*+1; (v) *x*, *y*−1, *z*; (vi) *x*−1, −*y*−1/2, *z*−1/2; (vii) −*x*−1, *y*−1/2, −*z*+1/2; (viii) −*x*−1, *y*+1/2, −*z*+1/2; (ix) *x*+1, −*y*−1/2, *z*+1/2.

### Hydrogen-bond geometry (Å, °)

**Table:**

*D*—H···*A*	*D*—H	H···*A*	*D*···*A*	*D*—H···*A*
O18—H18···O11	0.82	1.77	2.502 (3)	148
C3—H3···O11	0.93	2.39	2.758 (3)	103
C13—H13···O18^v^	0.93	2.56	3.282 (3)	135

Symmetry codes: (v) *x*, *y*−1, *z*.
